# Synthesis and Antihypertensive Screening of New Derivatives of Quinazolines Linked with Isoxazole

**DOI:** 10.1155/2014/739056

**Published:** 2014-06-12

**Authors:** Mujeeb Ur Rahman, Ankita Rathore, Anees A. Siddiqui, Gazala Parveen, M. Shahar Yar

**Affiliations:** ^1^Department of Pharmaceutical Chemistry, Faculty of Pharmacy, Jamia Hamdard (Hamdard University), Hamdard Nagar, New Delhi 110062, India; ^2^SunRise University, Alwar 301030, India

## Abstract

A series of 7-substituted-3-(4-(3-(4-substitutedphenyl)-4,5-dihydroisoxazol-5-yl)phenyl)-2-substituted quinazolin-4(3*H*)-one **(1–30)** have been synthesized by the cyclization of (*E*)-3-(4-(3-substitutedphenyl)acrylolyl)phenyl)-2-(substitutedphenyl)-7-substituted quinazolin-4-(3*H*)-one with hydroxylamine hydrochloride. The synthesized compounds were examined for their *in vivo* antihypertensive activity using albino rats. All the titled compounds exhibited good to moderate antihypertensive activity. Compounds 7-Chloro-3-(4-(3-(4-chlorophenyl)-4,5- dihydroisoxazol-5-yl)phenyl)-2-p-tolylquinazolin-4(*3H*)-one **(23)** and 7-Chloro-3-(4-(3-(4-chlorophenyl)-4,5-dihydroisoxazol-5-yl)phenyl)-2-(4-methoxyphenyl)quinazolin-4(*3H*)-one **(24)** exhibited potent antihypertensive activity through their anticipated **α**_1_-adrenergic receptor blocking property similar to its clinically used analogue, prazosin, without affecting heart rate with prolonged duration of action when tested in adrenaline induced hypertension in anaesthetized rats.

## 1. Introduction

Among the major risk factors for arterial diseases, hypertension is one of the most life threatening health problems in the modern world [1, 2]. In 2000, the figure of total number of adults with hypertension was 972 million and this may rise by about 60% to a total of 1.56 billion by 2025 [3]. Moreover, in India, about 70% of coronary heart disease-related deaths occur in people younger than 70 years [4]. *α*_1_-Adrenergic receptor antagonist like prazosin [5, 6], terazosin, and doxazosin are reputed class of antihypertensive agents derived from quinazoline. Prazosin has selectivity to blocks postsynaptic *α*_1_-adrenergic receptors while having no effect on presynaptic *α*_2_-adrenergic receptor and is responsible for the inhibition of norepinephrine release from sympathetic nerve, and the piperazine ring is also very sensitive towards enzymatic hydroxylation, whereas doxazosin and terazosin [7] were found to possess longer duration and less reflex tachycardia than prazosin. Since quinazoline derivatives, which belong to the *N*-containing heterocyclic compounds, have universal concerns due to their widely and distinct biological activities such as diuretic [8–10], antihypertensive [11], antihistaminic [12, 13] analgesic, and anti-inflammatory [14, 15] anticancer [16] and anti-HIV [17] activities, it was therefore worthwhile to study the possibility of replacing or changing in position of the labile furoylpiperazine moiety in prazosin by a more stable isoxazole group so that the antihypertensive activity of the new derivatives remains unaltered but possesses longer duration of action due to the increased stability against enzymatic degradation. In present study we have reported 30 molecules containing quinazoline and isoxazole heterocyclic ring.

**Figure 1:**
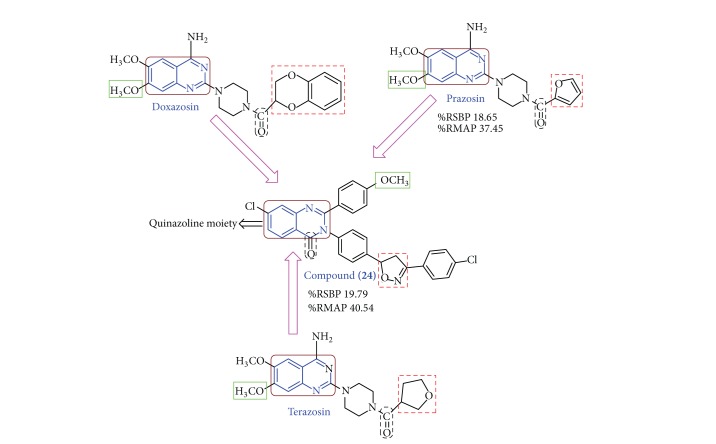
Chemical structure of active chemotherapeutic antihypertensive agents (prazosin, terazosin, and doxazosin) and rationally designed template for targeted compound (**24**). RSBP (rise in systolic blood pressure), RMAP (rise in mean atrial blood pressure).

Considering the above facts and in addition to our work on antihypertensive [18], we have synthesized 7-substituted-3-(4-(3-(4-substitutedphenyl)-4,5-dihydroisoxazol-5-yl)phenyl)-2-substituted quinazolin-4(3*H*)-one derivatives possessing antihypertensive activity with minimum side effects (Figure 1).

## 2. Materials

All the chemicals used were of laboratory grade and procured from E. Merck (Darmstadt, Germany) and S.D. Fine Chemicals (Mumbai, India). Melting points were determined by open capillary tubes in a Hicon melting point apparatus and are uncorrected. Purity of the compounds was checked by thin layer chromatography (TLC) plates (silica gel G). Iodine chamber and UV lamp were used for the visualization of TLC spots. The Fourier Transform Infrared (FT-IR) spectra were recorded on Bio-rad FTS-135 spectrophotometer using KBr pellets; *ν*_max⁡_ values are given in cm^−1^. The ^1^H NMR spectra were taken on a Bruker 400 Ultrashield (300/400 MHz) NMR spectrometers using dimethylsulfoxide (DMSO)-d6 solvent and tetramethylsilane (TMS) as an internal standard (chemical shift in *δ*, ppm). ^13^C NMR spectra were recorded on a Bruker 400 Ultrashield (400 MHz) NMR spectrometers using dimethylsulfoxide (DMSO)-d6 solvent. Mass spectra recorded on UPLC-MS/MS (water, Q-TOF-ESI, and Mass Lyns v 4.1) mass serial no. JAA-272 (Synapt MS-G_1_) were presented as *m*/*z*. Elemental analysis was carried out on CHN elemental (Perkin Elmer 240 analyser) using sulphanilic acid as a standard and tungsten (VI) oxide as a combusting agent. All the results of elemental analyses corresponded to the calculated values within experimental error. The reaction was monitored by thin-layer chromatography (TLC) and spots were visualized by iodine vapors or irradiation with UV light (254 nm).

## 3. Methods

### 3.1. Chemistry

#### 3.1.1. Substituted 2-Benzamidobenzoic Acid (**a1–a12**)

To the substituted anthranilic acid (2 mmol) dissolved in 10% sodium hydroxide (10 mL), substituted benzoyl chloride (2.2 mmol) was added with stirring at room temperature for over 1 h. Upon completion, reaction mixture was quenched with cold water to obtain solid residue, which was washed with dilute HCl followed by water and recrystallized from ethanol. Yield: 90–96%.

#### 3.1.2. 2-(Substituted) Phenyl-(substituted)-4H-benzo[d]oxazin-4-one (**b1–b12**)

A solution of substituted 2-benzamidobenzoic acid (**a1–a12**, 2 mmol) in acetic anhydride (10 mL) was heated under reflux for 2 h and then poured into crushed ice. The solid residue thus obtained was filtered, dried, and recrystallized with ethanol; Yield is 85–90%.

#### 3.1.3. 3-(4-Acetylphenyl)-7-substituted-2-(substitutedphenyl)quinazolin-4(3H)-one (**c1–c12**)

A mixture of substituted benzoxazine (**b1–b12**, 2 mmol) and* p*-aminoacetophenone (2 mmol) in methanol was heated under reflux for 3 h. The separated solid was collected by filtration, washed with water, dried, and recrystallized from ethanol.

#### 3.1.4. (E)-3-(4-(3-Substitutedphenyl)acrylolyl)phenyl)-2-(substitutedphenyl)-7-substituted quinazolin-4-(3H)-one (**d1–d30**)

To a magnetically stirred solution of substituted quinazolinone (**c1–c12**, 0.01 mmol) in ethanol, substituted aldehyde (0.01 mmol) was added and the reaction mixture was stirred for 2 h. Upon completion of reaction, the mixture was poured on crushed ice and then acidified with dilute HCl. A precipitate formed which was filtered, washed with water, and recrystallized with ethanol.

#### 3.1.5. 7-Substituted-3-(4-(3-(4-substitutedphenyl)-4,5-dihydroisoxazol-5-yl)phenyl)-2-substituted quinazolin-4(3H)-one (**1–30**)

A mixture of (*E*)-3-(4-(3-substitutedphenyl)acrylolyl)phenyl)-2-(substitutedphenyl)-7-substituted quinazolin-4-(3*H*)-one (**d1–d30**, 0.01 mol) in absolute ethanol (25 mL) and water; hydroxylamine hydrochloride (0.01 mol) was added. The reaction mixture was heated under reflux for 5 h in the presence of 30% KOH. The mixture was poured in ice water and then acidified with dilute HCl. The resulting solid was filtered, washed with water, dried, and crystallized with ethanol. 

*(1) 2-Phenyl-3-(4-(3-phenyl-4,5-dihydroisoxazol-5-yl)phenyl)quinazolin-4(3H)-one* **(1)**. Yield: 78%; m.p. 138–140°C; TLC solvent (T : E : F, 5 : 4 : 1); *R*_*f*_ 0.63; IR (KBr) *ν*_max⁡_ (cm^−1^): 3052 (Ar-CH), 1695 (CO), 1630 (C=N), 1608, 1455 (C=C), 1220 (C–O–N), 1115 (C–N); ^1^H NMR (DMSO-d6) *δ* (ppm); 3.80 (d, 2H,* J* = 8.8 Hz, CH_2isox._), 5.88 (dd, 1H,* J* = 5.5, 8.4 Hz, CH_isox._), 8.04 (d, 2H,* J* = 9.4 Hz, Ar-H), 8.06 (d, 4H,* J* = 7.0 Hz, Ar-H), 8.10 (d, 2H,* J* = 6.8 Hz, Ar-H), 8.12 (t, 2H,* J* = 5.2 Hz,* J* = 3.6 Hz, Ar-H), 8.14 (d, 2H,* J* = 8.8 Hz, Ar-H), 8.16 (d, 2H,* J* = 10 Hz, Ar-H), 8.15–8.17 (m, 4H, Ar-H); ^13^C NMR (DMSO-d6) *δ*; 128.5 (2C), 128.8 (2C), 131.8, 132.5 (phenyl), 43.7, 85.5, 155.7 (3C, isoxazole), 128.5 (2C), 128.8 (2C), 130.2, 131.3 (Ar-C), 124.4 (2C), 128.5 (2C), 132.45, 137.66 (Ar-C), 120.6, 126.6, 128.2, 133.8, 135.2, 148.3, 156.9 (quinazoline), 160.4 (1C, C=O); (*m*/*z*): 443.49 (M^+^); % Anal. Cal. for C_29_H_21_N_3_O_2_: C: 78.54, H: 4.77, N: 9.47. Found: C: 78.50, H: 4.72, N: 9.46.

*(2) 2-(2-Chlorophenyl)-3-(4-(3-Phenyl-4,5-dihydroisoxazol-5-yl)phenyl)quinazolin-4(3H)-one* ***(2)***. Yield: 84%; m.p. 140–142°C; TLC solvent (T : E : F, 5 : 4 : 1); *R*_*f*_ 0.66; IR (KBr, cm^−1^): 3022 (Ar-CH), 1685 (CO), 1610 (C=N), 1598, 1463 (C=C), 1230 (C–O–N), 1110 (C–N), 710 (C–Cl); ^1^H NMR (DMSO-*d*_6_) *δ*; 3.90 (d, 2H,* J* = 7.6 Hz, CH_2isox._), 6.02 (dd, 1H,* J* = 6.8, 2.8 Hz, CH_isox._), 8.06 (d, 2H,* J* = 11.6 Hz, Ar-H), 8.06–8.09 (m, 3H, Ar-H), 8.10 (d, 2H,* J* = 2.8 Hz, Ar-H), 8.12 (t, 2H,* J* = 3.2,* J* = 5.6 Hz, Ar-H), 8.14 (d, 2H,* J* = 7.2 Hz, Ar-H), 8.15 (d, 2H,* J* = 11.6 Hz, Ar-H), 8.16–8.18 (m, 4H, Ar-H);^ 13^C NMR (DMSO-*d*_6_) *δ*; 128.2 (2C), 129.2 (2C), 130.8, 131.2 (phenyl), 42.2, 83.4, 156.5 (3C, isoxazole), 123.5, 126.5, 128.8, 130.2, 131.3, 132.3 (Ar-C), 125.8 (2C), 128.2 (2C), 132.5, 138.3 (Ar-C), 120.4, 127.7, 128.8, 134.2, 135.6, 147.6, 155.4 (quinazoline), 162.3 (1C, C=O); % Anal. Cal. for C_29_H_20_ClN_3_O_2_; C, 72.88; N, 8.79; H, 4.22. Found; C, 72.86; N, 8.77; H, 4.20; Ms (*m*/*z*): 477.94 (M^+^).

*(3) 3-(4-(3-Phenyl-4,5-dihydroisoxazol-5-yl)phenyl)-2-p-tolylquinazolin-4(3H)-one* **(3)**. Yield: 70%; m.p. 162–164°C; TLC solvent (T : E : F, 5 : 4 : 1); *R*_*f*_ 0.63; IR (KBr, cm^−1^): 3012 (Ar-CH), 1690 (CO), 1634 (C=N), 1618, 1465 (C=C), 1225 (C–O–N), 1123 (C–N); ^1^H NMR (DMSO-d6) *δ* (ppm); 2.23 (s, 3H, CH_3_), 3.92 (d, 2H,* J* = 8.4 Hz, CH_2isox._), 6.00 (dd, 1H,* J* = 2.8, 7.2 Hz, CH_isox._), 8.03–8.06 (m, 5H, Ar-H), 8.08 (d, 2H,* J* = 4 Hz, Ar-H), 8.08-8.09 (m, 2H, Ar-H), 8.12 (d, 2H,* J* = 7.6 Hz, Ar-H), 8.15 (d, 2H,* J* = 8.4 Hz, Ar-H), 8.16-8.17 (m, 4H, Ar-H); ^13^C NMR (DMSO-d6) *δ*; 20.5 (1C, CH_3_), 128.5 (2C), 128.8 (2C), 131.0, 132.6 (phenyl), 40.8, 84.7, 157.2 (3C, isoxazole), 125.2, 129.5 (2C), 130.8 (2C), 140.6 (Ar-C), 126.2 (2C), 128.8 (2C), 131.2, 137.5 (Ar-C), 120.8, 126.4, 128.8, 133.6, 136.3, 146.5, 156.4 (quinazoline), 160.6 (1C, C=O); % Anal. Cal. for C_30_H_23_N_3_O_2_; C, 78.75; N, 9.18; H, 5.07. Found; C, 78.76; N, 9.18; H, 5.05; Ms (*m*/*z*): 457.52 (M^+^).

*(4) 2-(4-Methoxyphenyl)-3-(4-(3-phenyl-4,5-dihydroisoxazol-5-yl)phenyl)quinazolin-4(3H)-one* ***(4)***. Yield: 65%; m.p. 135–137°C; TLC solvent (T : E : F, 5 : 4 : 1); *R*_*f*_ 0.67; IR (KBr, cm^−1^): 3022 (Ar-CH), 1675 (CO), 1625 (C=N), 1588, 1425 (C=C), 1310 (C–O), 1226 (C–O–N), 1110 (C–N); ^1^H NMR (DMSO-d6) *δ* (ppm); 2.70 (s, 3H, OCH_3_), 3.80 (d, 2H,* J* = 8.0 Hz, CH_2isox._), 5.78 (d, 1H,* J* = 12.4 Hz, CH_isox._), 8.02–8.04 (m, 5H, Ar-H), 8.07 (d, 2H,* J* = 8.4 Hz, Ar-H), 8.09 (d, 2H,* J* = 3.2 Hz, Ar-H), 8.11–8.20 (m, 8H, Ar-H); ^13^C NMR (DMSO-d6) *δ*; 52.6 (1C, OCH_3_), 128.5 (2C), 129.8 (2C), 130.2, 131.5 (phenyl), 42.0, 84.2, 155.7 (3C, isoxazole), 118.4 (2C), 120.5, 132.4 (2C), 158.4 (Ar-C), 124.4 (2C), 126.8 (2C), 131.4, 138.0 (Ar-C), 120.8, 127.6, 128.2, 133.6, 135.6, 148.3, 156.0 (quinazoline), 160.2 (1C, C=O); % Anal. Cal. for C_30_H_23_N_3_O_3_; C, 76.09; N, 8.87; H, 4.90. Found; C, 76.08; N, 8.87; H, 4.92; Ms (*m*/*z*): 473.52 (M^+^).

*(5) 7-Chloro-2-phenyl-3-(4-(3-phenyl-4,5-dihydroisoxazol-5-yl)phenyl)quinazolin-4(3H)-one* ***(5)***. Yield: 80%; m.p. 166–168°C; TLC solvent (T : E : F, 5 : 4 : 1); *R*_*f*_ 0.54; IR (KBr, cm^−1^): 3062 (Ar-CH), 1680 (CO), 1650 (C=N), 1618, 1465 (C=C), 1226 (C–O–N), 1122 (C–N), 720 (C–Cl); ^1^H NMR (DMSO-d6) *δ* (ppm); 3.83 (d, 2H,* J* = 6.6 Hz, CH_2isox._), 5.88 (dd, 1H,* J* = 4.5,* J* = 7.7 Hz, CH_isox._), 7.98–8.00 (m, 5H, Ar-H), 8.04 (d, 2H,* J* = 5.5 Hz, Ar-H), 8.06 (d, 2H,* J* = 8.8 Hz, Ar-H), 8.08 (d, 2H,* J* = 9.4 Hz, Ar-H), 8.12 (d, 2H,* J* = 3.8 Hz, Ar-H), 8.14–8.16 (m, 4H, Ar-H); ^13^C NMR (DMSO-d6) *δ*; 128.5 (2C), 129.0 (2C), 131.4, 132.5 (phenyl), 42.6, 84.6, 155.0 (3C, isoxazole), 128.4 (2C), 128.6, 128.8 (2C), 132.5 (Ar-C), 125.2 (2C), 128.6 (2C), 131.0, 135.8 (Ar-C), 118.5, 122.5, 127.6, 130.8, 137.8, 152.4, 156.2 (quinazoline), 161.5 (1C, C=O); % Anal. Cal. for C_29_H_20_ClN_3_O_2_; C, 72.88; N, 8.79; H, 4.22. Found; C, 72.86; N, 8.77; H, 4.20; Ms (*m*/*z*): 478.94 (M+1).

*(6) 7-Chloro-2-(2-chlorophenyl)-3-(4-(3-phenyl-4,5-dihydroisoxazol-5-yl)phenyl)quinazolin-4(3H)-one* ***(6)***. Yield: 85%; m.p. 130–132°C; TLC solvent (T : E : F, 5 : 4 : 1); *R*_*f*_ 0.65; IR (KBr, cm^−1^): 3034 (Ar-CH), 1710 (CO), 1660 (C=N), 1598, 1480 (C=C), 1224 (C–O–N), 1176 (C–N), 750, 610 (C–Cl); ^1^H NMR (DMSO-d6) *δ* (ppm); 3.94 (d, 2H,* J* = 6.9 Hz, CH_2isox._), 5.86 (dd, 1H,* J* = 1.2, 6.9 Hz, CH_isox._), 8.03–8.06 (m, 5H, Ar-H), 8.06–8.08 (m, 3H, Ar-H), 8.10 (d, 1H,* J* = 5.4 Hz, Ar-H), 8.14 (d, 2H,* J* = 0.6 Hz, Ar-H), 8.17 (d, 2H,* J* = 3.6 Hz, Ar-H), 8.20–8.22 (m, 3H, Ar-H); ^13^C NMR (DMSO-d6) *δ*; 128.2 (2C), 130.8 (2C), 131.4, 132.3 (phenyl), 45.7, 88.5, 154.7 (3C, isoxazole), 120.6, 124.8, 130.2, 132.3 (2C), 134.5 (Ar-C), 125.8 (2C), 127.8 (2C), 132.4, 137.6 (Ar-C), 118.3, 120.6, 128.2, 130.8, 138.25, 151.3, 155.9 (quinazoline), 162.4 (1C, C=O); % Anal. Cal. for C_29_H_19_Cl_2_N_3_O_2_; C, 67.98; N, 8.20; H, 3.74. Found; C, 67.96; N, 8.22; H, 3.72; Ms (*m*/*z*): 512.38 (M+1). 

*(7) 7-Chloro-3-(4-(3-phenyl-4,5-dihydroisoxazol-5-yl)phenyl)-2-p-tolylquinazolin-4(3H)-one* ***(7)***. Yield: 70%; m.p. 164–166°C; TLC solvent (B : A, 9 : 1); *R*_*f*_ 0.67; IR (KBr, cm^−1^): 3012 (Ar-CH), 1690 (CO), 1644 (C=N), 1602, 1445 (C=C), 1225 (C–O–N), 1154 (C–N), 720 (C–Cl); ^1^H NMR (DMSO-d6) *δ* (ppm); 2.32 (s, 3H, CH_3_), 3.96 (d, 2H,* J* = 6.7 Hz, CH_2isox._), 5.80 (dd, 1H,* J* = 7.8, 7.0 Hz, CH_isox._), 8.00–8.02 (m, 5H, Ar-H), 8.04 (d, 2H,* J* = 7.8 Hz, Ar-H), 8.07 (d, 2H,* J* = 9.2 Hz, Ar-H), 8.10 (d, 2H,* J* = 8.4 Hz, Ar-H), 8.12 (d, 2H,* J* = 9.2 Hz, Ar-H), 8.11–8.18 (m, 3H, Ar-H); ^13^C NMR (DMSO-d6) *δ*; 20.4 (1C, CH_3_), 128.5 (2C), 129.8 (2C), 130.2, 131.8 (phenyl), 42.6, 84.2, 154.3 (3C, isoxazole), 125.5, 128.5 (2C), 130.3 (2C), 138.9 (Ar-C), 125.2 (2C), 127.5 (2C), 131.8, 137.2 (Ar-C), 118.8, 122.6, 130.6, 134.8, 137.4, 153.4, 156.7 (quinazoline), 160.2 (1C, C=O); % Anal. Cal. for C_30_H_22_ClN_3_O_2_; C, 73.24; N, 8.54; H, 4.51. Found; C, 73.23; N, 8.52; H, 4.50; Ms (*m*/*z*): 491.96 (M^+^).

*(8) 7-Chloro-2-(4-methoxyphenyl)-3-(4-(3-phenyl-4,5-dihydroisoxazol-5-yl)phenyl)quinazolin-4(3H)-one* ***(8)***. Yield: 85%; m.p. 170–172°C; TLC solvent (B : A, 9 : 1); *R*_*f*_ 0.70; IR (KBr, cm^−1^): 3022 (Ar-CH), 1693 (CO), 1610 (C=N), 1588, 1463 (C=C), 1295 (C–O), 1233 (C–O–N), 1122 (C–N), 722 (C–Cl); ^1^H NMR (DMSO-d6) *δ* (ppm); 2.75 (s, 3H, OCH_3_), 3.94 (d, 2H,* J* = 6.9 Hz, CH_2isox._), 5.82 (dd, 1H,* J* = 2.8,* J* = 8.0 Hz, CH_isox._), 8.04–8.06 (m, 5H, Ar-H), 8.08 (d, 2H,* J* = 8.8 Hz, Ar-H), 8.10 (d, 2H,* J* = 6.6 Hz, Ar-H), 8.14 (d, 2H,* J* = 7.6 Hz, Ar-H), 8.18 (d, 2H,* J* = 4.4 Hz, Ar-H), 8.20–8.22 (m, 3H, Ar-H); ^13^C NMR (DMSO-d6) *δ*; 52.5 (1C, OCH_3_), 128.2 (2C), 128.8 (2C), 130.6, 132.5 (phenyl), 42.0, 85.6, 155.6 (3C, isoxazole), 125.8, 127.4 (2C), 132.3 (2C), 139.4 (Ar-C), 125.2 (2C), 127.5 (2C), 131.8, 137.2 (Ar-C), 118.2, 120.5, 131.4, 133.4, 136.8, 152.5, 157.3 (quinazoline), 162.5 (1C, C=O); % Anal. Cal. for C_30_H_22_ClN_3_O_3_; C, 70.93; N, 8.27; H, 4.37. Found; C, 70.92; N, 8.25; H, 4.35; Ms (*m*/*z*): 507.96 (M+1).

*(9) 7-Methyl-2-phenyl-3-(4-(3-phenyl-4,5-dihydroisoxazol-5-yl)phenyl)quinazolin-4(3H)-one* ***(9)***. Yield: 55%; m.p. 133–135°C; TLC solvent (B : A, 9 : 1); *R*_*f*_ 0.80; IR (KBr, cm^−1^): 3012 (Ar-CH), 1690 (CO), 1610 (C=N), 1588, 1420 (C=C), 1232 (C–O–N), 1100 (C–N); ^1^H NMR (DMSO-d6) *δ* (ppm); 2.30 (s, 3H, CH_3_), 3.97 (d, 2H,* J* = 4.5 Hz, CH_2isox._), 5.86 (d, 1H,* J* = 7.8 Hz, CH_isox._), 7.94–8.00 (m, 5H, Ar-H), 8.02–8.04 (m, 3H, Ar-H), 8.07 (t, 1H,* J* = 2.8, 6.0 Hz, Ar-H), 8.10 (d, 2H,* J* = 6.9 Hz, Ar-H), 8.13 (d, 2H,* J* = 7.7 Hz, Ar-H), 8.17–8.20 (m, 4H, Ar-H); ^13^C NMR (DMSO-d6) *δ*; 21.5 (1C, CH_3_), 128.2 (2C), 128.8 (2C), 131.5, 132.5 (phenyl), 42.0, 83.2, 153.5 (3C, isoxazole), 124.3, 128.8 (2C), 131.2 (2C), 132.3 (Ar-C), 125.8 (2C), 128.2 (2C), 130.8, 138.0 (Ar-C), 118.5, 120.6, 132.4, 133.8, 142.0, 151.2, 156.7 (quinazoline), 162.4 (1C, C=O); % Anal. Cal. for C_30_H_23_N_3_O_2_; C, 78.75; N, 9.18; H, 5.07. Found; C, 78.76; N, 9.16; H, 5.05; Ms (*m*/*z*): 457.52 (M^+^).

*(10) 2-(2-Chlorophenyl)-7-methyl-3-(4-(3-phenyl-4,5-dihydroisoxazol-5-yl)phenyl)quinazolin-4(3H)-one* ***(10)***. Yield: 60%; m.p. 154–156°C; TLC solvent (T : E : F, 5 : 4 : 1); *R*_*f*_ 0.71; IR (KBr, cm^−1^): 3002 (Ar-CH), 1710 (CO), 1624 (C=N), 1608, 1488 (C=C), 1220 (C–O–N), 1105 (C–N), 710 (C–Cl); ^1^H NMR (DMSO-*d*_6_) *δ*: 2.22 (s, 3H, CH_3_), 3.87 (d, 2H, CH_2isox._), 5.81 (dd, 1H, CH_isox._), 8.02–7.98 (m, 5H, Ar-H), 8.04–8.02 (d, 2H, Ar-H), 8.07–8.03 (d, 2H, Ar-H), 8.10–8.08 (d, 2H, Ar-H), 8.16–8.13 (d, 2H, Ar-H), 8.22–8.19 (m, 3H, Ar-H);^ 13^C NMR (DMSO-*d*_6_) *δ*; 13.46 (1C, CH_3_), 106.16, 108.65, 110.54, 114.20, 122.62, 121.13 (6C, phenyl), 63.44, 150.12, 154.12 (3C, isoxazole), 115.82, 122.13, 134.12, 134.84, 138.12, 154.25 (6C, Ar_2_-C), 108.16, 115.44, 118.65, 126.52, 127.06, 133.24 (6C, Ar_1_-C), 108.65, 110.58, 115.17, 126.52, 150.53, 147.62, 160.62 (7C, quinazoline), 171.66 (1C, C=O); % Anal. Cal. for C_30_H_22_ClN_3_O_2_; C, 73.24; N, 8.54; H, 4.51. Found; C, 73.22; N, 8.52; H, 4.50; Ms (*m*/*z*): 492.96 (M+1).

*(11) 7-Methyl-3-(4-(3-phenyl-4,5-dihydroisoxazol-5-yl)phenyl)-2-p-tolylquinazolin-4(3H)-one* ***(11)***. Yield: 66%; m.p. 178–180°C; TLC solvent (T : E : F, 5 : 4 : 1); *R*_*f*_ 0.58; IR (KBr, cm^−1^): 3012 (Ar-CH), 1678 (CO), 1612 (C=N), 1572, 1453 (C=C), 1218 (C–O–N), 1112 (C–N); ^1^H NMR (DMSO-d6) *δ* (ppm); 2.21 (s, 3H, CH_3_), 2.36 (s, 3H, CH_3_), 3.80 (d, 2H,* J* = 2.1 Hz, CH_2isox._), 5.74 (dd, 1H,* J* = 4.5, 2.4 Hz, CH_isox._), 8.04–8.08 (m, 5H, Ar-H), 8.10 (d, 2H,* J* = 3.3 Hz, Ar-H), 8.12 (d, 2H,* J* = 4.5 Hz, Ar-H), 8.15 (d, 2H,* J* = 4.5 Hz, Ar-H), 8.22 (d, 2H,* J* = 7.2 Hz, Ar-H), 8.30–8.32 (m, 3H, Ar-H); ^13^C NMR (DMSO-d6) *δ*; 21.6 (2C, CH_3_), 128.0 (2C), 128.6 (2C), 131.2, 132.5 (phenyl), 42.2, 85.5, 153.6 (3C, isoxazole), 124.2, 127.6 (2C), 130.7 (2C), 139.2 (Ar-C), 125.8 (2C), 128.3 (2C), 130.8, 136.2 (Ar-C), 118.8, 120.3, 130.6, 133.2, 143.2, 152.4, 157.4 (quinazoline), 160.4 (1C, C=O); % Anal. Cal. for C_31_H_25_N_3_O_2_; C, 78.96; N, 8.91; H, 5.34. Found; C, 78.97; N, 8.90; H, 5.31; Ms (*m*/*z*): 471.54 (M^+^).

*(12) 2-(4-Methoxyphenyl)-7-methyl-3-(4-(3-phenyl-4,5-dihydroisoxazol-5-yl)phenyl)quinazolin-4(3H)-one* ***(12)***. Yield: 76%; m.p. 172–174°C; TLC solvent (T : E : F, 5 : 4 : 1); *R*_*f*_ 0.58; IR (KBr, cm^−1^): 3012 (Ar-CH), 1677 (CO), 1612 (C=N), 1596, 1453 (C=C), 1300 (C–O), 1226 (C–O–N), 1106 (C–N); ^1^H NMR (DMSO-d6) *δ* (ppm); 2.28 (s, 3H, CH_3_), 2.82 (s, 3H, OCH_3_), 3.86 (d, 2H,* J* = 7.0 Hz, CH_2isox._), 5.89 (m, 1H, CH_isox._), 7.96–7.94 (m, 5H, Ar-H), 8.08 (d, 2H,* J* = 7.5 Hz, Ar-H), 8.14 (d, 2H,* J* = 7.4 Hz, Ar-H), 8.18 (d, 2H,* J* = 8.4 Hz, Ar-H), 8.20 (d, 2H,* J* = 6.6 Hz, Ar-H), 8.21–8.23 (m, 3H, Ar-H); ^13^C NMR (DMSO-d6) *δ*; 20.4 (1C, CH_3_), 50.5 (1C, OCH_3_) 128.2 (2C), 128.9 (2C), 131.6, 132.5 (phenyl), 42.0, 84.8, 153.5 (3C, isoxazole), 125.2, 128.0 (2C), 131.4 (2C), 138.5 (Ar-C), 125.6 (2C), 128.0 (2C), 132.4, 138.2 (Ar-C), 120.8, 122.6, 130.6, 133.4, 138.0, 151.4, 155.7 (quinazoline), 161.5 (1C, C=O); % Anal. Cal. for C_31_H_25_N_3_O_3_; C, 76.37; N, 8.62; H, 5.17. Found; C, 76.32; N, 8.60; H, 5.15; Ms (*m*/*z*): 488.54 (M+1).

*(13) 3-(4-(3-(4-Chlorophenyl)-4, 5-dihydroisoxazol-5-yl)phenyl)-2-phenylquinazolin-4(3H)-one* ***(13)***. Yield: 70%; m.p. 145–147°C; TLC solvent (B : A, 9 : 1); *R*_*f*_ 0.70; IR (KBr, cm^−1^): 3012 (Ar-CH), 1684 (CO), 1618 (C=N), 1596, 1473 (C=C), 1222 (C–O–N), 1102 (C–N), 712 (C–Cl); ^1^H NMR (DMSO-d6) *δ* (ppm); 3.55 (d, 2H,* J* = 7.5 Hz, CH_2isox._), 5.92 (m, 1H, CH_isox._), 8.06–8.08 (m, 4H, Ar-H), 8.07–8.09 (m, 4H, Ar-H), 8.10 (d, 2H,* J* = 3.3 Hz, Ar-H), 8.12 (d, 2H,* J* = 6.6 Hz, Ar-H), 8.18–8.20 (m, 5H, Ar-H); ^13^C NMR (DMSO-d6) *δ*; 128.0 (2C), 128.2, 129.2 (2C), 137.2 (phenyl), 42.3, 85.2, 155.6 (3C, isoxazole), 127.2, 128.4 (2C), 128.8 (2C), 130.2 (Ar-C), 125.8 (2C), 128.2 (2C), 130.2, 138.0 (Ar-C), 120.4, 123.5, 130.3, 135.3, 138.0, 148.6, 155.7 (quinazoline), 160.5 (1C, C=O); % Anal. Cal. for C_29_H_20_ClN_3_O_2_; C, 72.88; N, 8.79; H, 4.22. Found; C, 72.87; N, 8.75; H, 4.20; Ms (*m*/*z*): 478.94 (M+1). 

*(14) 2-(2-Chlorophenyl)-3-(4-(3-(4-chlorophenyl)-4,5-dihydroisoxazol-5-yl)phenyl)quinazolin-4(3H)-one* ***(14)***. Yield: 76%; m.p. 150–152°C; TLC solvent (T : E : F, 5 : 4 : 1); *R*_*f*_ 0.66; IR (KBr, cm^−1^): 3033 (Ar-CH), 1698 (CO), 1622 (C=N), 1590, 1473 (C=C), 1227 (C–O–N), 1132 (C–N), 752 (C–Cl); ^1^H NMR (DMSO-d6) *δ* (ppm); 3.74 (d, 2H,* J* = 7.8 Hz, CH_2isox._), 5.92 (d, 1H,* J* = 2.8 Hz, CH_isox._), 8.03–8.05 (m, 4H, Ar-H), 8.06–8.08 (m, 4H, Ar-H), 8.12 (d, 2H,* J* = 8.2 Hz, Ar-H), 8.13 (d, 2H,* J* = 6.8 Hz, Ar-H), 8.38–8.40 (m, 4H, Ar-H); ^13^C NMR (DMSO-d6) *δ*; 128.2 (2C), 129.8 (2C), 130.8, 137.4 (phenyl), 43.3, 84.6, 156.6 (3C, isoxazole), 126.3, 128.2 (2C), 128.8 (2C), 138.0 (Ar-C), 122.2, 126.8, 130.2, 131.6, 132.0, 132.6 (Ar-C), 120.8, 124.6, 132.6, 133.8, 137.4, 148.4, 155.7 (quinazoline), 160.5 (1C, C=O); % Anal. Cal. for C_29_H_19_Cl_2_N_3_O_2_; C, 67.98; N, 8.20; H, 3.74. Found; C, 67.96; N, 8.18; H, 3.73; Ms (*m*/*z*): 515 (M+2). 

*(15) 3-(4-(3-(4-Chlorophenyl)-4,5-dihydroisoxazol-5-yl)phenyl)-2-p-tolylquinazolin-4(3H)-one* ***(15)***. Yield: 77%; m.p. 145–147°C; TLC solvent (T : E : F, 5 : 4 : 1); *R*_*f*_ 0.67; IR (KBr, cm^−1^): 3038 (Ar-CH), 1710 (CO), 1605 (C=N), 1590, 1453 (C=C), 1226 (C–O–N), 1117 (C–N), 733 (C–Cl); ^1^H NMR (DMSO-d6) *δ* (ppm); 2.21 (s, 3H, CH_3_), 3.68 (d, 2H,* J* = 9.2 Hz, CH_2isox._), 5.86 (m, 1H, CH_isox._), 8.06–8.08 (m, 4H, Ar-H), 8.12–8.14 (m, 4H, Ar-H), 8.18 (d, 2H,* J* = 8.2 Hz, Ar-H), 8.19 (d, 2H,* J* = 7.9 Hz, Ar-H), 8.42–8.44 (m, 4H, Ar-H); ^13^C NMR (DMSO-d6) *δ*; 21.8 (1C, CH_3_), 128.2 (2C), 128.8 (2C), 131.2, 137.9 (phenyl), 42.2, 83.8, 155.6 (3C, isoxazole), 125.6, 129.2 (2C), 130.5 (2C), 139.2 (Ar-C), 125.8 (2C), 128.5 (2C), 130.8, 138.2 (Ar-C), 120.8, 122.5, 130.3, 133.6, 134.4, 147.4, 157.7 (quinazoline), 162.4 (1C, C=O); % Anal. Cal. for C_30_H_22_ClN_3_O_2_; C, 73.24; N, 8.24; H, 4.51. Found; C, 73.22; N, 8.22; H, 4.52; Ms (*m*/*z*): 492.96 (M+1). 

*(16) 3-(4-(3-(4-Chlorophenyl)-4,5-dihydroisoxazol-5-yl) phenyl)-2-(4-methoxyphenyl) quinazolin-4(3H)-one* ***(16)***. Yield: 66%; m.p. 164–166°C; TLC solvent (T : E : F, 5 : 4 : 1); *R*_*f*_ 0.68; IR (KBr, cm^−1^): 3010 (Ar-CH), 1696 (CO), 1622 (C=N), 1598, 1466 (C=C), 1285 (C–O), 1217 (C–O–N), 1120 (C–N), 717 (C–Cl); ^1^H NMR (DMSO-d6) *δ* (ppm); 2.78 (s, 3H, OCH_3_), 3.96 (d, 2H,* J* = 7.6 Hz, CH_2isox._), 5.92 (m, 1H, CH_isox._), 8.04–8.02 (m, 4H, Ar-H), 8.04–8.06 (m, 4H, Ar-H), 8.08 (d, 2H,* J* = 4.8 Hz, Ar-H), 8.10 (d, 2H,* J* = 9.2 Hz, Ar-H), 8.18–8.20 (m, 4H, Ar-H); ^13^C NMR (DMSO-d6) *δ*; 52.6 (1C, OCH_3_), 128.6 (2C), 129.2, 130.5 (2C), 137.9 (phenyl), 40.6, 84.3, 156.2 (3C, isoxazole), 115.6 (2C), 121.6, 130.8 (2C), 162.8 (Ar-C), 124.2 (2C), 127.9 (2C), 130.2, 137.8 (Ar-C), 120.2, 126.4, 128.6, 129.2, 133.5, 147.8, 156.2 (quinazoline), 160.2 (1C, C=O); % Anal. Cal. for C_30_H_22_ClN_3_O_3_; C, 70.93; N, 8.27; H, 4.37. Found; C, 70.91; N, 8.25; H, 4.35; Ms (*m*/*z*): 508.96 (M+1). 

*(17) 3-(4-(3-(4-Methoxyphenyl)-4,5-dihydroisoxazol-5-yl)phenyl)-2-phenylquinazolin-4(3H)-one* ***(17)***. Yield: 60%; m.p. 190–192°C; TLC solvent (T : E : F, 5 : 4 : 1); *R*_*f*_ 0.70; IR (KBr, cm^−1^): 3018 (Ar-CH), 1713 (CO), 1634 (C=N), 1586, 1453 (C=C), 1296 (C–O), 1234 (C–O–N), 1110 (C–N); ^1^H NMR (DMSO-d6) *δ* (ppm); 2.74 (s, 3H, OCH_3_), 3.68 (d, 2H,* J* = 2.9 Hz, CH_2isox._), 5.92 (d, 1H,* J* = 8.4 Hz, CH_isox._), 8.00–8.02 (m, 4H, Ar-H), 8.04–8.06 (m, 4H, Ar-H), 8.15 (d, 2H,* J* = 8.3 Hz, Ar-H), 8.16 (d, 2H,* J* = 7.4 Hz, Ar-H), 8.38–8.40 (m, 5H, Ar-H); ^13^C NMR (DMSO-d6) *δ*; 54.6 (1C, OCH_3_), 115.8 (2C), 122.5, 127.3 (2C), 160.6 (phenyl), 40.8, 84.2, 156.4 (3C, isoxazole), 127.2 (2C), 128.2 (2C), 128.8, 130.6 (Ar-C), 126.8 (2C), 127.8 (2C), 131.4, 137.5 (Ar-C), 120.4, 126.5, 127.3, 129.4, 134.8, 148.0, 155.4 (quinazoline), 161.2 (1C, C=O); % Anal. Cal. for C_30_H_23_N_3_O_3_; C, 76.09; N, 8.87; H, 4.90. Found; C, 76.07; N, 8.85; H, 4.92; Ms (*m*/*z*): 475.52 (M+2). 

*(18) 2-(2-Chlorophenyl)-3-(4-(3-(4-methoxyphenyl)-4,5-dihydroisoxazol-5-yl)phenyl)quinazolin-4(3H)-one* ***(18)***. Yield: 70%; m.p. 165–167°C; TLC solvent (B : A, 9 : 1); *R*_*f*_ 0.63; IR (KBr, cm^−1^): 3018 (Ar-CH), 1713 (CO), 1634 (C=N), 1586, 1453 (C=C), 1308 (C–O), 1234 (C–O–N), 1110 (C–N); ^1^H NMR (DMSO-d6) *δ* (ppm); 2.68 (s, 3H, OCH_3_), 3.88 (d, 2H,* J* = 6.4 Hz, CH_2isox._), 5.80 (m, 1H, CH_isox._), 8.06–8.08 (m, 4H, Ar-H), 8.10–8.12 (m, 4H, Ar-H), 8.15 (d, 2H,* J* = 7.8 Hz, Ar-H), 8.30 (d, 2H,* J* = 8.0 Hz, Ar-H), 8.38–8.40 (m, 4H, Ar-H); ^13^C NMR (DMSO-d6) *δ*; 55.2 (1C, OCH_3_), 114.5 (2C), 123.5, 128.8 (2C), 161.2 (phenyl), 42.4, 83.8, 157.4 (3C, isoxazole), 123.6, 126.8, 129.2, 130.4, 131.8, 132.5 (Ar-C), 125.6 (2C), 128.2 (2C), 130.4, 138.5 (Ar-C), 120.4, 126.2, 126.8, 127.9, 133.8, 147.5, 154.5 (quinazoline), 160.5 (1C, C=O); % Anal. Cal. for C_30_H_22_ClN_3_O_3_; C, 70.93; N, 8.27; H, 4.37. Found; C, 70.95; N, 8.25; H, 4.35; Ms (*m*/*z*): 508.96 (M+1). 

*(19) 3-(4-(3-(4-Methoxyphenyl)-4,5-dihydroisoxazol-5-yl) phenyl)-2-p-tolylquinazolin-4(3H)-one* ***(19)***. Yield: 73%; m.p. 176–178°C; TLC solvent (B : A, 9 : 1); *R*_*f*_ 0.60; IR (KBr, cm^−1^): 3015 (Ar-CH), 1697 (CO), 1618 (C=N), 1588, 1468 (C=C), 1280 (C–O), 1230 (C–O–N), 1154 (C–N); ^1^H NMR (DMSO-d6) *δ* (ppm); 2.03 (s, 3H, CH_3_), 2.56 (s, 3H, OCH_3_), 3.80 (d, 2H,* J* = 8.8 Hz, CH_2isox._), 5.88 (d, 1H,* J* = 9.2 Hz, CH_isox._), 8.04 (d, 2H,* J* = 9.2 Hz, Ar-H), 8.06 (d, 2H,* J* = 6.4 Hz, Ar-H), 8.10 (d, 2H,* J* = 3.6 Hz, Ar-H), 8.12 (t, 2H,* J* = 8.0, 7.4 Hz, Ar-H), 8.14 (d, 2H,* J* = 4.4 Hz, Ar-H), 8.16 (d, 2H,* J* = 8.4, Ar-H), 8.15–8.17 (m, 4H, Ar-H); ^13^C NMR (DMSO-d6) *δ*; 20.4 (1C, CH_3_), 55.3 (1C, OCH_3_), 114.4 (2C), 120.3, 128.8 (2C), 161.5 (phenyl), 42.2, 83.0, 155.6 (3C, isoxazole), 125.2, 129.5 (2C), 130.3 (2C), 140.2 (Ar-C), 125.2 (2C), 128.2 (2C), 130.4, 138.2 (Ar-C), 120.4, 126.2, 127.8, 130.4, 133.8, 148.8, 156.2 (quinazoline), 160.8 (1C, C=O); % Anal. Cal. for C_31_H_25_N_3_O_3_; C, 76.37; N, 8.62; H, 5.17. Found; C, 76.35; N, 8.60; H, 5.19; Ms (*m*/*z*): 487.54 (M^+^).

*(20) 2-(4-Methoxyphenyl)-3-(4-(3-(4-methoxyphenyl)-4,5-dihydroisoxazol-5-yl)phenyl) quinazolin-4(3H)-one* ***(20)***. Yield: 65%; m.p. 162–164°C; TLC solvent (T : E : F, 5 : 4 : 1); *R*_*f*_ 0.55; IR (KBr, cm^−1^): 3024 (Ar-CH), 1688 (CO), 1612 (C=N), 1595, 1472 (C=C), 1298 (C–O), 1224 (C–O–N), 1160 (C–N); ^1^H NMR (DMSO-d6) *δ* (ppm); 2.80 (s, 3H, OCH_3_), 2.86 (s, 3H, OCH_3_), 3.84 (d, 2H,* J* = 5.8 Hz, CH_2isox._), 5.92 (m, 1H, CH_isox._), 8.08–8.10 (m, 4H, Ar-H), 8.12–8.14 (m, 4H, Ar-H), 8.15 (d, 2H,* J* = 8.0 Hz, Ar-H), 8.17 (d, 2H,* J* = 9.4 Hz, Ar-H), 8.20–8.22 (m, 4H, Ar-H); ^13^C NMR (DMSO-d6) *δ*; 54.7(2C, OCH_3_), 116.2 (2C), 122.8, 127.2 (2C), 162.3 (phenyl), 42.5, 84.5, 156.2 (3C, isoxazole), 115.6 (2C), 120.3, 130.3 (2C), 160.8 (Ar-C), 125.8 (2C), 129.5 (2C), 131.5, 137.5 (Ar-C), 120.3, 126.6, 128.3, 130.8, 132.5, 147.3, 157.5 (quinazoline), 162.3 (1C, C=O); % Anal. Cal. for C_31_H_25_N_3_O_4_; C, 73.94; N, 8.34; H, 5.00. Found; C, 73.92; N, 8.32; H, 5.02; Ms (*m*/*z*): 503.54 (M^+^).

*(21) 7-Chloro-3-(4-(3-(4-chlorophenyl)-4,5-dihydroisoxazol-5-yl)phenyl)-2-phenylquinazolin-4(3H)-one* ***(21)***. Yield: 68%; m.p. 180–182°C; TLC solvent (T : E : F, 5 : 4 : 1); *R*_*f*_ 0.54; IR (KBr, cm^−1^): 3032 (Ar-CH), 1684 (CO), 1608 (C=N), 1592, 1426 (C=C), 1228 (C–O–N), 1124 (C–N), 712 (C–Cl); ^1^H NMR (DMSO-d6) *δ* (ppm); 3.93 (d, 2H,* J* = 7.8 Hz, CH_2isox._), 5.95 (d, 1H,* J* = 8.3 Hz, CH_isox._), 8.10–8.12 (m, 4H, Ar-H), 8.14–8.16 (m, 3H, Ar-H), 8.20 (d, 2H,* J* = 8.4 Hz, Ar-H), 8.22 (d, 2H,* J* = 7.2 Hz, Ar-H), 8.28–8.32 (m, 5H, Ar-H); ^13^C NMR (DMSO-d6) *δ*; 128.2 (2C), 128.8, 129.2 (2C), 137.2 (phenyl), 41.5, 85.0, 156.2 (3C, isoxazole), 128.2 (2C), 128.6, 129.5 (2C), 130.7 (Ar-C), 125.5 (2C), 127.8 (2C), 131.4, 137.6 (Ar-C), 118.4, 125.2, 126.8, 132.4, 137.5, 152.2, 157.5 (quinazoline), 160.8 (1C, C=O); % Anal. Cal. for C_29_H_19_Cl_2_N_3_O_2_; C, 67.98; N, 8.20; H, 3.74. Found; C, 67.96; N, 8.17; H, 3.72; Ms (*m*/*z*): 513.38 (M+1). 

*(22) 7-Chloro-2-(2-chlorophenyl)-3-(4-(3-(4-chlorophenyl)-4,5-dihydroisoxazol-5-yl)phenyl)quinazolin-4(3H)-one* ***(22)***. Yield: 75%; m.p. 157–159°C; TLC solvent (T : E : F, 5 : 4 : 1); *R*_*f*_ 0.67; IR (KBr, cm^−1^): 3042 (Ar-CH), 1688 (CO), 1628 (C=N), 1587, 1420 (C=C), 1225 (C–O–N), 1113 (C–N), 734 (C–Cl); ^1^H NMR (DMSO-d6) *δ* (ppm); 3.84 (d, 2H,* J* = 5.4 Hz, CH_2isox._), 5.90 (m, 1H, CH_isox._), 8.10–8.12 (m, 4H, Ar-H), 8.12–8.14 (m, 3H, Ar-H), 8.16 (d, 2H,* J* = 7.8 Hz, Ar-H), 8.18 (d, 2H,* J* = 9.4 Hz, Ar-H), 8.18–8.20 (m, 4H, Ar-H); ^13^C NMR (DMSO-d6) *δ*; 118.4 (2C), 122.6, 128.2 (2C), 136.2 (phenyl), 42.5, 84.0, 157.6 (3C, isoxazole), 123.5, 126.5, 130.3, 131.2, 132.3, 132.8 (Ar-C), 124.8 (2C), 128.6 (2C), 130.2, 137.5 (Ar-C), 118.4, 122.2, 125.6, 129.4, 137.5, 150.8, 157.4 (quinazoline), 162.4 (1C, C=O); % Anal. Cal. for C_29_H_18_Cl_3_N_3_O_2_; C, 63.70; N, 7.68; H, 3.32. Found; C, 63.72; N, 7.66; H, 3.30; Ms (*m*/*z*): 547.83 (M+1). 

*(23) 7-Chloro-3-(4-(3-(4-chlorophenyl)-4,5-dihydroisoxazol-5-yl)phenyl)-2-p-tolylquinazolin-4(3H)-one* ***(23)***. Yield: 74%; m.p. 181–183°C; TLC solvent (T : E : F, 5 : 4 : 1); *R*_*f*_ 0.70; IR (KBr, cm^−1^): 3038 (Ar-CH), 1694 (CO), 1622 (C=N), 1595, 1420 (C=C), 1305 (C–O), 1216 (C–O–N), 1114 (C–N), 710 (C–Cl); ^1^H NMR (DMSO-d6) *δ* (ppm); 2.23 (s, 3H, CH_3_), 3.76 (d, 2H,* J* = 7.2 Hz, CH_2isox._), 5.86 (d, 1H,* J* = 10.4 Hz, CH_isox._), 7.96–7.98 (m, 4H, Ar-H), 8.02–8.04 (m, 4H, Ar-H), 8.06–8.08 (m, 4H, Ar-H), 8.16 (d, 2H,* J* = 8.8 Hz, Ar-H), 8.20 (d, 2H,* J* = 8.0 Hz, Ar-H); ^13^C NMR (DMSO-d6) *δ*; 22.5 (1C, CH_3_), 128.2 (2C), 128.6, 128.8 (2C), 135.8 (phenyl), 43.0, 83.5, 156.2 (3C, isoxazole), 125.8, 129.0 (2C), 130.8 (2C), 138.8 (Ar-C), 125.8 (2C), 127.8 (2C), 131.2, 138.0 (Ar-C), 118.8, 122.7, 127.5, 130.6, 137.4, 152.5, 156.4 (quinazoline), 162.3 (1C, C=O); % Anal. Cal. for C_30_H_21_Cl_2_N_3_O_2_; C, 68.45; N, 7.98; H, 4.02. Found; C, 68.47; N, 7.96; H, 4.00; Ms (*m*/*z*): 528.41 (M+2). 

*(24) 7-Chloro-3-(4-(3-(4-chlorophenyl)-4,5-dihydroisoxazol-5-yl)phenyl)-2-(4-methoxyphenyl)quinazolin-4(3H)-one* ***(24)***. Yield: 80%; m.p. 186–188°C; TLC solvent (B : A, 9 : 1); *R*_*f*_ 0.69; IR (KBr, cm^−1^): 3036 (Ar-CH), 1680 (CO), 1622 (C=N), 1596, 1420 (C=C), 1300 (C–O), 1233 (C–O–N), 1130 (C–N), 735 (C–Cl); ^1^H NMR (DMSO-d6) *δ* (ppm); 2.65 (s, 3H, OCH_3_), 3.84 (d, 2H,* J* = 8.0 Hz, CH_2isox._), 6.01 (m, 1H, CH_isox._), 7.98–8.00 (m, 4H, Ar-H), 8.04–8.06 (m, 3H, Ar-H), 8.08–8.10 (m, 4H, Ar-H), 8.14 (d, 2H,* J* = 8.0 Hz, Ar-H), 8.18 (d, 2H,* J* = 4.8 Hz, Ar-H); ^13^C NMR (DMSO-d6) *δ*; 52.4 (1C, OCH_3_), 128.3 (2C), 128.5, 128.9 (2C), 136.4 (phenyl), 43.8, 82.6, 158.6 (3C, isoxazole), 115.5 (2C), 120.8, 131.5 (2C), 162.4 (Ar-C), 125.2 (2C), 128.5 (2C), 132.6, 138.5 (Ar-C), 118.2, 120.5, 128.3, 131.4, 137.8, 151.4, 157.3 (quinazoline), 160.2 (1C, C=O); % Anal. Cal. for C_30_H_21_Cl_2_N_3_O_3_; C, 66.43; N, 7.75; H, 3.90. Found; C, 66.41; N, 7.73; H, 3.88; Ms (*m*/*z*): 544.11 (M+2). 

*(25) 3-(4-(3-(4-Chlorophenyl)-4,5-dihydroisoxazol-5-yl)phenyl)-7-methyl-2-phenylquinazolin-4(3H)-one* ***(25)***. Yield: 64%; m.p. 150–152°C; TLC solvent (B : A, 9 : 1); *R*_*f*_ 0.62; IR (KBr, cm^−1^): 3028 (Ar-CH), 1692 (CO), 1618 (C=N), 1590, 1422 (C=C), 1222 (C–O–N), 1120 (C–N); ^1^H NMR (DMSO-d6) *δ* (ppm); 2.12 (s, 3H, CH_3_), 3.80 (d, 2H,* J* = 7.2 Hz, CH_2isox._), 5.88 (d, 1H,* J* = 4.8 Hz, CH_isox._), 7.92–7.94 (m, 3H, Ar-H), 8.08–8.10 (m, 5H, Ar-H), 8.12 (d, 2H,* J* = 10.2 Hz, Ar-H), 8.14 (d, 2H,* J* = 9.2 Hz, Ar-H), 8.33–8.35 (m, 4H, Ar-H); ^13^C NMR (DMSO-d6) *δ*; 22.5 (1C, CH_3_), 128.2 (2C), 128.6, 128.8 (2C), 135.8 (phenyl), 43.0, 83.5, 156.2 (3C, isoxazole), 125.8, 129.0 (2C), 130.8 (2C), 138.8 (Ar-C), 125.8 (2C), 127.8 (2C), 131.2, 138.0 (Ar-C), 118.8, 122.7, 127.5, 130.6, 137.4, 152.5, 156.4 (quinazoline), 162.3 (1C, C=O); % Anal. Cal. for C_30_H_22_ClN_3_O_2_; C, 73.24; N, 8.54; H, 4.51. Found; C, 73.22; N, 8.52; H, 4.50; Ms (*m*/*z*): 491.96 (M^+^).

*(26) 2-(2-Chlorophenyl)-3-(4-(3-(4-chlorophenyl)-4,5-dihydroisoxazol-5-yl)phenyl)-7-methylquinazolin-4(3H)-one* ***(26)***. Yield: 73%; m.p. 185–187°C; TLC solvent (B : A, 9 : 1); *R*_*f*_ 0.54; IR (KBr, cm^−1^): 3040 (Ar-CH), 1682 (CO), 1622 (C=N), 1596, 1424 (C=C), 1232 (C–O–N), 1112 (C–N), 720 (C–Cl); ^1^H NMR (DMSO-d6) *δ* (ppm); 2.34 (s, 3H, CH_3_), 3.89 (d, 2H,* J* = 8.2 Hz, CH_2isox._), 5.93(m, 1H, CH_isox._), 7.94–7.96 (m, 3H, Ar-H), 8.10 (d, 2H,* J* = 11.2 Hz, Ar-H), 8.13 (d, 2H,* J* = 2.8 Hz, Ar-H), 8.14–8.16 (m, 4H, Ar-H), 8.20–8.24 (m, 4H, Ar-H); ^13^C NMR (DMSO-d6) *δ*; 20.8 (1C, CH_3_), 128.6 (2C), 129.6, 130.2 (2C), 136.2 (phenyl), 42.6, 84.2, 155.8 (3C, isoxazole), 123.4, 126.6, 130.4, 131.8, 132.2, 132.8 (Ar-C), 124.5 (2C), 126.2 (2C), 130.6, 137.6 (Ar-C), 118.8, 124.7, 128.5, 132.6, 143.5, 151.0, 157.2 (quinazoline), 162.3 (1C, C=O); % Anal. Cal. for C_30_H_21_Cl_2_N_3_O_2_; C, 68.45; N, 7.98; H, 4.02. Found; C, 68.43; N, 7.96; H, 4.00; Ms (*m*/*z*): 528.41 (M+2). 

*(27) 3-(4-(3-(4-Chlorophenyl)-4, 5-dihydroisoxazol-5-yl)phenyl)-7-methyl-2-p-tolylquinazolin-4(3H)-one* ***(27)***. Yield: 64%; m.p. 176–178°C; TLC solvent (B : A, 9 : 1); *R*_*f*_ 0.74; IR (KBr, cm^−1^): 3030 (Ar-CH), 1692 (CO), 1614 (C=N), 1588, 1420 (C=C), 1225 (C–O–N), 1130 (C–N), 708 (C–Cl); ^1^H NMR (DMSO-d6) *δ* (ppm); 2.20 (s, 3H, CH_3_), 2.24 (s, 3H, CH_3_), 3.84 (d, 2H,* J* = 2.8 Hz, CH_2isox._), 5.90 (d, 1H,* J* = 8.2 Hz, CH_isox._), 7.88–7.90 (m, 3H, Ar-H), 7.97-7.98 (m, 4H, Ar-H), 8.10 (d, 2H,* J* = 6.4 Hz, Ar-H), 8.12 (d, 2H,* J* = 8.2 Hz, Ar-H), 8.16–8.18 (m, 4H, Ar-H); ^13^C NMR (DMSO-d6) *δ*; 22.5 (2C, CH_3_), 128.0 (2C), 128.8, 129.2 (2C), 136.0 (phenyl), 42.8, 84.2, 156.6 (3C, isoxazole), 125.8, 129.2 (2C), 130.8 (2C), 140.2 (Ar-C), 125.2 (2C), 128.5 (2C), 130.4, 138.8 (Ar-C), 118.2, 120.7, 128.5, 130.2, 142.4, 151.2, 155.6 (quinazoline), 160.5 (1C, C=O); % Anal. Cal. for C_31_H_24_ClN_3_O_2_; C, 73.58; N, 8.30; H, 4.78. Found; C, 73.56; N, 8.28; H, 4.76; Ms (*m*/*z*): 506.99 (M+1). 

*(28) 3-(4-(3-(4-Cchlorophenyl)-4, 5-dihydroisoxazol-5-yl) phenyl)-2-(4-methoxyphenyl)-7-methylquinazolin-4(3H)-one* ***(28)***. Yield: 86%; m.p. 122–124°C; TLC solvent (B : A, 9 : 1); *R*_*f*_ 0.62; IR (KBr, cm^−1^): 3036 (Ar-CH), 1687 (CO), 1624 (C=N), 1586, 1416 (C=C), 1288 (C–O), 1224 (C–O–N), 1104 (C–N), 732 (C–Cl); ^1^H NMR (DMSO-d6) *δ* (ppm); 2.19 (s, 3H, CH_3_), 2.96 (s, 3H, OCH_3_), 3.96 (d, 2H,* J* = 8.0 Hz, CH_2isox._), 5.78 (m, 1H, CH_isox._), 8.00–8.02 (m, 3H, Ar-H), 8.04–8.06 (m, 4H, Ar-H), 8.08 (d, 2H,* J* = 5.2 Hz, Ar-H), 8.12 (d, 2H,* J* = 8.0 Hz, Ar-H), 8.13–8.15 (m, 4H, Ar-H); ^13^C NMR (DMSO-d6) *δ*; 20.2 (1C, CH_3_), 53.8 (1C, OCH_3_), 128.2 (2C), 128.7 129.8 (2C), 135.5 (phenyl), 42.4, 85.7, 155.8 (3C, isoxazole), 115.2 (2C), 120.2, 131.2 (2C), 162.5 (Ar-C), 125.6 (2C), 128.8 (2C), 131.4, 137.4 (Ar-C), 117.0, 124.7, 127.5, 130.5, 143.5, 150.2, 156.2 (quinazoline), 161.8 (1C, C=O); % Anal. Cal. for C_31_H_24_ClN_3_O_3_; C, 71.33; N, 8.05; H, 4.63. Found; C, 71.31; N, 8.03; H, 4.65; Ms (*m*/*z*): 521.99 (M^+^).

*(29) 7-Chloro-3-(4-(3-(4-methoxyphenyl)-4,5-dihydroisoxazol-5-yl)phenyl)-2-phenylquinazolin-4(3H)-one* ***(29)***. Yield: 65%; m.p. 190–192°C; TLC solvent (B : A, 9 : 1); *R*_*f*_ 0.75; IR (KBr, cm^−1^): 3040 (Ar-CH), 1680 (CO), 1614 (C=N), 1595, 1417 (C=C), 1308 (C–O), 1230 (C–O–N), 1120 (C–N), 722 (C–Cl); ^1^H NMR (DMSO-d6) *δ* (ppm); 2.78 (s, 3H, OCH_3_), 3.85 (d, 2H,* J* = 4.6 Hz, CH_2isox._), 5.80 (d, 1H,* J* = 8.2 Hz, CH_isox._), 8.06–8.08 (m, 4H, Ar-H), 8.10 (d, 2H,* J* = 8.8 Hz, Ar-H), 8.12 (d, 2H,* J* = 6.0 Hz, Ar-H), 8.13–8.15 (m, 3H, Ar-H), 8.16–8.18 (m, 5H, Ar-H); ^13^C NMR (DMSO-d6) *δ*; 52.0 (1C, OCH_3_), 115.5 (2C), 122.6 128.2 (2C), 160.8 (phenyl), 43.2, 84.7, 156.4 (3C, isoxazole), 128.2 (2C), 128.6, 129.5 (2C), 130.2 (Ar-C), 124.5 (2C), 128.3 (2C), 130.4, 138.2 (Ar-C), 118.6, 122.6, 127.8, 130.5, 138.2, 152.8, 157.7 (quinazoline), 160.4 (1C, C=O); % Anal. Cal. for C_30_H_22_ClN_3_O_3_; C, 70.93; N, 8.27; H, 4.37. Found; C, 70.95; N, 8.25; H, 4.35; Ms (*m*/*z*): 507.96 (M^+^).

*(30) 7-Chloro-2-(2-chlorophenyl)-3-(4-(3-(4-methoxyphenyl)-4, 5-dihydroisoxazol-5-yl)phenyl)quinazolin-4(3H)-one* ***(30)***. Yield: 86%; m.p. 179–181°C; TLC solvent (B : A, 9 : 1); *R*_*f*_ 0.64; IR (KBr, cm^−1^): 3035 (Ar-CH), 1696 (CO), 1620 (C=N), 1598, 1430 (C=C), 1278 (C–O), 1234 (C–O–N), 1134 (C–N), 722 (C–Cl); ^1^H NMR (DMSO-d6) *δ* (ppm); 2.82 (s, 3H, OCH_3_), 3.83 (d, 2H,* J* = 8.4 Hz, CH_2isox._), 5.94 (m, 1H, CH_isox._), 7.96–7.98 (m, 4H, Ar-H), 8.00–8.02 (m, 3H, Ar-H), 8.02–8.04 (m, 4H, Ar-H), 8.08 (d, 2H,* J* = 6.2 Hz, Ar-H), 8.10 (d, 2H,* J* = 9.4 Hz, Ar-H); ^13^C NMR (DMSO-d6) *δ*; 55.9 (1C, OCH_3_), 116.4 (2C), 122.5 128.2 (2C), 160.4(phenyl), 43.4, 84.6, 156.2 (3C, isoxazole), 122.5, 126.8, 130.5, 131.6, 132.2, 132.8 (Ar-C), 125.0 (2C), 127.6 (2C), 131.8, 138.9 (Ar-C), 118.6, 122.5, 128.2, 131.5, 137.8, 151.6, 155.8 (quinazoline), 160.2 (1C, C=O); % Anal. Cal. for C_30_H_21_Cl_2_N_3_O_3_; C, 66.43; N, 7.75; H, 3.90. Found; C, 66.41; N, 7.73; H, 3.92; Ms (*m*/*z*): 544.41 (M+2).

### 3.2. Pharmacology

All the experimental protocols were carried out with the permission from the Institutional Animal Ethics committee (IAEC), project proposal no. 781 and the guidelines provided by the Committee for the Purpose of Control and Supervision of Experiments in Animal (CPCSEA). Animals were obtained from the Central Animal House Facility, Hamdard University, New Delhi. Registration number and date of registration are 173/CPCSEA, 28th of January, 2000. All rats were housed in a temperature and humidity controlled room at an ambient temperature of 25 ± 2°C with a 12 h light/dark cycle. Animals were provided with pellets diet (Lipton, Calcutta, India) and water ad libitum.

#### 3.2.1. Antihypertensive Activity

#### 3.2.2. Conditioning/Training of Animals

For conducting the BP measurement studies, the animals were kept in restrainers for 10 min every day for one week. This exercise was done to avoid the fluctuation in blood pressure due to aggressive behaviour of the animal.

#### 3.2.3. Induction of Hypertension in Albino Rats

After recording the initial BP of rats, the animals were divided into groups of 6 animals each. One group was taken as control. Hypertension was induced in the remaining groups by subcutaneous injection of methyl prednisolone acetate (20 mg/kg body wt./wk) for 2 weeks as per method reported by Krakoff et al. [19].

#### 3.2.4. Dose Establishment of Synthesized Compounds for Screening of Antihypertensive Activity

Systolic blood pressure was measured in conscious rats using CODA noninvasive blood pressure recorder by Tail Cuff method (Kent Scientific Corporation, USA). The restrainers carrying the rat were placed in the BP instrument with the tail protruding out. The tail was gently placed in contact with a transducer membrane, which was connected to the digital BP display panel. The instrument was then turned on and allowed to stabilize until steady pulse rate was observed. Once the “pulse level ready” signal appeared, the BP recording button was pressed and the mean arterial BP was recorded. Albino rats (body weight 200–250 g) were used in the study. Rats were assigned to groups of six animals in each. Each compound (1 mg/day, 2 mg/day,…,10 mg/day) was administered orally after suspending in 1% carboxy methyl cellulose (CMC) solution. The blood pressure was recorded at various time intervals.

#### 3.2.5. Determination of *α*-Adrenergic Receptors Blocking Activity

Albino rats were classified into ten groups; each group comprises 6 animals each, to investigate the *α*_1_-adrenergic receptor blocking effect of some synthesized compounds. In each experiment, the effect of adrenaline, at a dose of 3 *μ*g/Kg intravenously, on the arterial blood pressure was recorded alone 30 min before i.p. injection (5 mg/kg b.w. for compounds** 1**,** 16**,** 19**,** 20**,** 23**,** 27**, and** 28,** whereas 4 mg/kg b.w. for compound** 24**) of the test compound and then its effect was determined again after 30, 60, and 120 min from the injection of the test compounds [20].

#### 3.2.6. Statistical Analysis of Data

The statistical analysis was performed using GRAPHPAD INSTAT 3 software (Graph Pad Software Inc., San Diego, CA). Data obtained from animal experiments were expressed as arithmetic mean ± SEM and ±S.E. The comparison between various groups was performed by one-way analysis of variance (ANOVA), and the effects in treatment groups were compared with toxic control or control group by Dunnett multiple comparison test. *P* < 0.05 was considered to be significant (*P < 0.05; **P < 0.01).

## 4. Result and Discussions

### 4.1. Chemistry

**Scheme 1:**
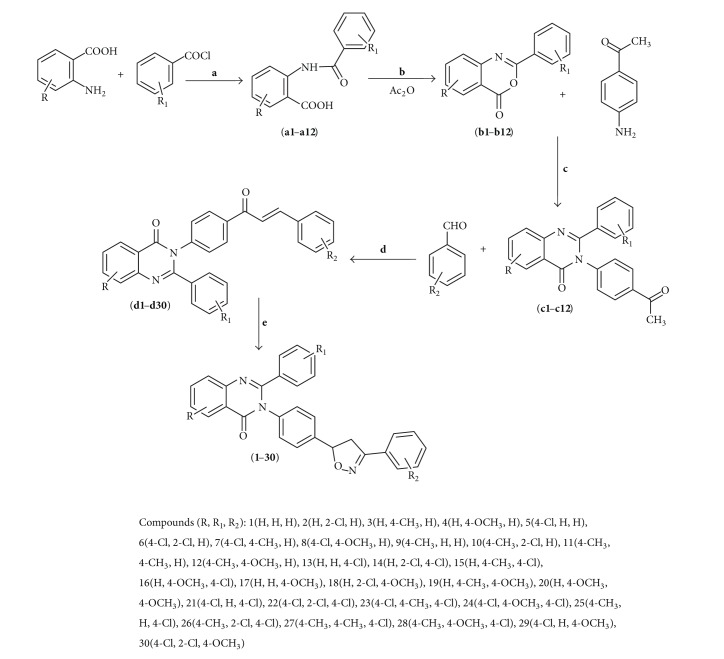
Synthesis of 7-substituted-3-(4-(3-(4-substitutedphenyl)-4,5-dihydroisoxazol-5-yl)phenyl)-2-substituted quinazolin-4(3*H*)-one. Reagents and conditions: (a) 10% NaOH, rt, 60 min; (b) reflex for 2 h; (c) methanol reflux for 3 h; (d) ethanol, NaOH, stirring at rt, 2 h; (e) NH_2_OH·HCl, 30% KOH, ethanol, dist. water, at water bath, 5 h.

7-Substituted-3-(4-(3-(4-substitutedphenyl)-4,5-dihydroisoxazol-5-yl)phenyl)-2-substituted quinazolin-4(3*H*)-one derivatives (**1–30**) were synthesized according to Scheme 1. Substituted anthranilic acids were reacted with substituted benzoyl chloride via stirring the reactants at 10% NaOH to yield substituted amides** (a1–a12)**. Some substituted benzoxazines** (b1–b12)** were obtained by heating** (a1–a12)** at reflux in acetic anhydride [21–25]. Substituted 3-(4-acetylphenyl)-2-phenylquinazolin-4(3*H*)-one** (c1–c12)** were yielded by the refluxing of substituted benzoxazines with* p*-aminoacetophenone [26, 27]. According to the Claisen-Schmidt condensation, substituted 3-(4-acetylphenyl)-2-phenylquinazolin-4(3*H*)-one with aromatic aldehydes in equimolar amount were stirred in ethanol for 2 h to yield different chalcones [28]. Finally, different chalcones and hydroxylamine hydrochloride were heated on a water bath in ethanol containing KOH to obtain title compounds (**1–30**) [29]. The purity of all the target compounds was ensured using thin-layer chromatography (TLC) in different solvent systems and melting point techniques and was further confirmed by both the analytical and spectral data of the proposed structures. For instance, for compound** 24**, The IR spectra of mentioned molecule showed characteristic absorption bands at 3036 cm^−1^ (Ar-CH), 1680 cm^−1^ (CO), 1622 cm^−1^ (C=N), 1596, 1420 cm^−1^ (C=C), 1300 cm^−1^, 1233 cm^−1^ (C–O–N), 1130 cm^−1^ (C–N), and 735 cm^−1^ (C–Cl). The ^1^H NMR spectra showed two doublet at *δ* 8.14 (2H,* J* = 8.0 Hz, Ar-H) and 8.18 (2H,* J *= 4.8 Hz, Ar-H) confirming N_2_-Ar ring, one doublet at *δ* 3.84 (d, 2H,* J* = 8.0 Hz, CH_2isox._) confirming CH_2 _isoxazole, and one multiplet at *δ* 6.01 confirming CH isoxazole. The multiplets at *δ* 8.00–7.98, 8.06–8.04, and 8.10–8.08 are indicatives of 4 protons of Ar_2_ ring, 3 protons of quinazoline moiety, and 4 protons of N_3_-Ar ring, respectively. The one singlet corresponding to OCH_3_ was obtained at *δ* 2.65. The mass spectrum shows the presence of molecular ion peak at *m*/*z* 544.11 (M+2) according to the molecular formula, C_30_H_21_Cl_2_N_3_O_3_. The structure was also supported by elemental analysis data which was ±0.4%. The other compounds are also characterized in a similar manner.

### 4.2. Antihypertensive Activity

#### 4.2.1. Dose Establishment for the Screening of Antihypertensive Activity

**Figure 2:**
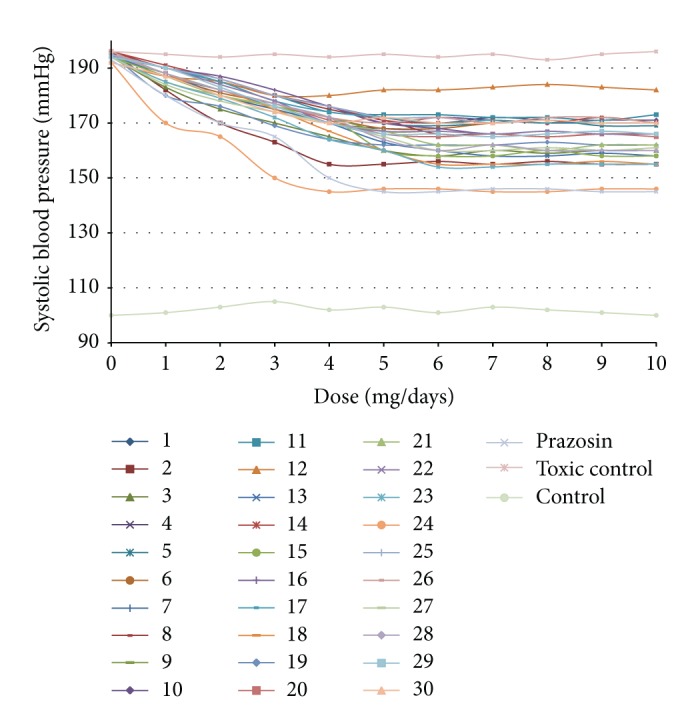
Graph showing dose establishment for the screening of antihypertensive activity.

The minimum effective dose of test compounds was established by performing the gradual enhanced dose administration (1 mg/day, 2 mg/day, 3 mg/day,…, 10 mg/day), and when there was no further decrease, that is, ceiling effect in the blood pressure, observed at a certain dose, that dose was assumed as the minimum dose required to elicit the optimum antihypertensive activity of the synthesised compound. All titled compounds were screened to study their effect on the arterial blood pressure by using noninvasive Tail Cuff method. The results are compared with standard drug prazosin [30], as shown in Table 1. Most of the synthetic compounds showed the ceiling dose between 5-6 mg. The title compounds** 2**,** 18**,** 19**,** 23,** and** 27** were found to show substantial reduction in systolic blood pressure, whereas compound** 24** showed significant antihypertensive activity as compared to standard drug prazosin (Figure 2). The ceiling dose of compound** 24** was found to be 4 mg; however, prazosin has a ceiling dose of 5 mg. The rest of the compounds showed moderate decrease in hypertension.

#### 4.2.2. Determination of *α*-Adrenergic Receptor Blocking Property

**Figure 3:**
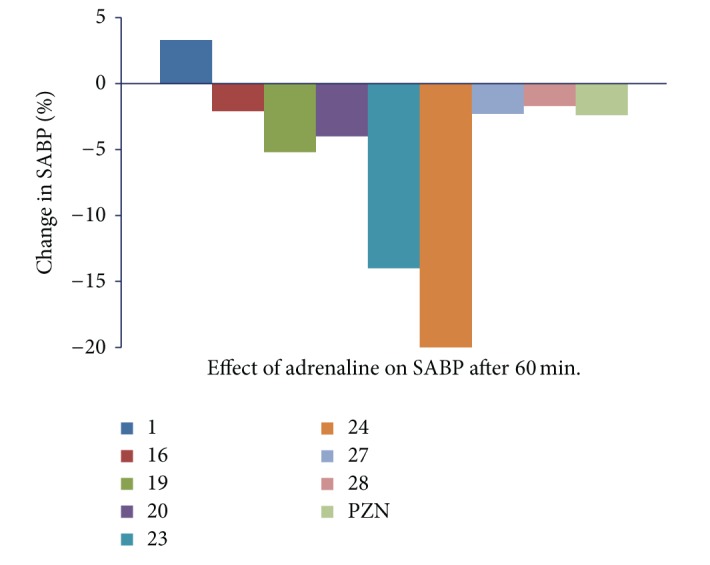
Graph showing effect of i.v. inj. of adrenaline (3 *μ*g/Kg) 30 min after prazosin and some test compounds** 1**,** 16**,** 19**,** 20**,** 23**,** 24**,** 27**, and** 28** at a dose of 5 mg/Kg on systolic blood pressure of anaesthetized albino rats.

**Figure 4:**
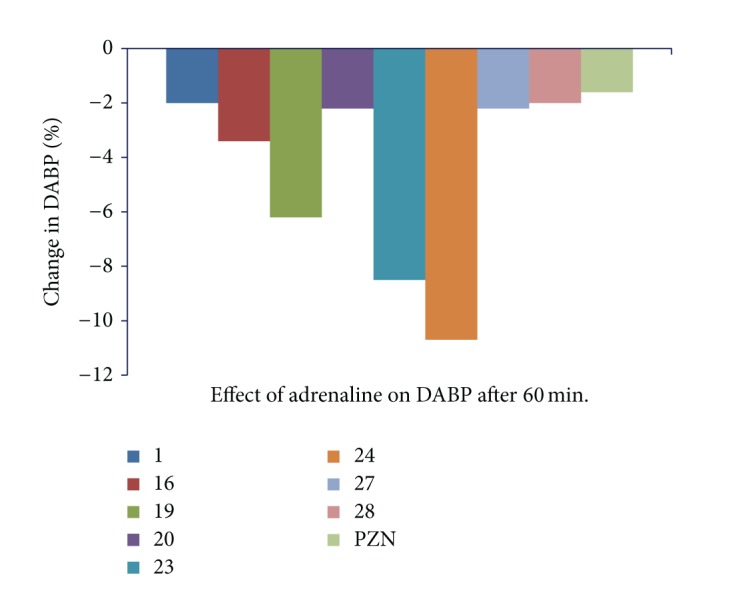
Graph showing effect of i.v. inj. of adrenaline (3 *μ*g/Kg) 30 min after prazosin and some test compounds** 1**,** 16**,** 19**,** 20**,** 23**,** 24**,** 27**, and** 28** at a dose of 5 mg/Kg on diastolic blood pressure of anaesthetized albino rats.

The injection of prazosin, 30 min prior to i.v injection of adrenaline at a dose of 3 *μ*g/Kg [31], produced a significant (*P* < 0.05) drop in arterial blood pressure due to selective blockade of *α*_1_-adrenergic receptor by prazosin (adrenaline antagonist) [11]; however, injection of prazosin, 60 min prior to adrenaline injection returned the blood pressure to normal level; this effect may be due to rapid onset and short duration of action of drug (Figures 3 and 4). Amongst the tested compounds, compound** 1** showed the significant (*P* < 0.05) increase in SABP and DABP and without changing heart rate at 30 min prior to administration of adrenaline. Therefore this compound could serve as lead molecule for the synthesis of a series of compounds to treat hypotension. Compounds** 23** and** 24** showed reverse action of vasopressor effect of adrenaline with depressor reflex after 30 min, as shown in Table 2. This effect was further prolonged at 60 min (Figures 3 and 4). The activity was continued up to 120 min to check the further change in activity. On the basis of these observations, it may be hypothesized that these compounds possess rapid and prolonged *α*_1_-adrenergic blocking property without causing reflex tachycardia. Compounds** 16**,** 27**, and** 28** also exhibited moderate antagonist activity of adrenaline after 30 min. It indicates that these compounds also possess *α*_1_-adrenergic receptor blocking effect with shorter duration of action like prazosin.

## 5. Conclusion

In conclusion, we have synthesized new quinazoline derivatives associated with isoxazole. These compounds showed significant antihypertensive activity with minimal side effects. The most potent *α*_1_-adrenergic blocking property was exhibited by the compound** 24**, having an SABP & DABP -11.6, −12.6 at 30 min. The replacement of piperazine moiety of prazosin at position-2 with substituted phenyl ring and incorporation of substituted isoxazole ring at position-3 of quinazoline moiety proved beneficial for antihypertensive activity. Our study shows that these synthesized compounds act as lead and may be useful in antihypertensive therapy.

## Figures and Tables

**Table 1:** Dose establishment for the screening of antihypertensive activity.

Compd.	Average systolic blood pressure (mmHg) with different oral doses (mg/day) at different time (days)											MAP (Mean ± SEM)	% Reduction in MAP	% Inhibition In SBP
	0	1	2	3	4	5	6	7	8	9	10			
1	192 ± 1	187 ± 2	185 ± 3	180 ± 2	180 ± 2	182 ± 3	182 ± 3	183 ± 2	184 ± 2	183 ± 2	182 ± 2	183 ± 1.03^ns^	11.09	04.68
2	196 ± 1	182 ± 2	170 ± 3	163 ± 2	158 ± 2	158 ± 3	156 ± 3	155 ± 2	156 ± 2	155 ± 2	155 ± 2	163 ± 4.12**	31.00	16.83
3	194 ± 1	183 ± 2	175 ± 3	170 ± 2	165 ± 2	160 ± 3	158 ± 3	160 ± 2	159 ± 2	160 ± 2	160 ± 2	167 ± 3.56**	27.09	13.91
4	195 ± 1	188 ± 2	182 ± 3	176 ± 2	170 ± 2	168 ± 3	168 ± 3	170 ± 2	171 ± 2	170 ± 2	170 ± 2	175 ± 2.75**	19.45	10.25
5	195 ± 1	190 ± 2	186 ± 3	180 ± 2	176 ± 2	170 ± 3	169 ± 3	170 ± 2	171 ± 2	169 ± 2	169 ± 2	176 ± 2.87**	17.90	09.74
6	192 ± 1	187 ± 2	181 ± 3	177 ± 2	172 ± 2	168 ± 3	168 ± 3	170 ± 2	171 ± 2	170 ± 2	170 ± 2	175 ± 2.47**	19.63	08.85
7	195 ± 1	188 ± 2	180 ± 3	175 ± 2	170 ± 2	165 ± 3	160 ± 3	160 ± 2	161 ± 2	160 ± 2	160 ± 2	170 ± 3.78**	24.36	12.82
8	196 ± 1	190 ± 2	186 ± 3	180 ± 2	175 ± 2	171 ± 3	166 ± 3	166 ± 2	165 ± 2	166 ± 2	166 ± 2	175 ± 3.37**	19.54	10.71
9	194 ± 1	188 ± 2	182 ± 3	176 ± 2	172 ± 2	167 ± 3	167 ± 3	166 ± 2	167 ± 2	166 ± 2	166 ± 2	173 ± 3.02**	21.00	10.82
10	195 ± 1	190 ± 2	185 ± 3	180 ± 2	176 ± 2	170 ± 3	172 ± 3	171 ± 2	170 ± 2	171 ± 2	171 ± 2	177 ± 2.69**	17.36	09.23
11	194 ± 1	190 ± 2	185 ± 3	180 ± 2	174 ± 2	173 ± 3	173 ± 3	172 ± 2	172 ± 2	171 ± 2	173 ± 2	177 ± 2.45**	16.81	08.76
12	194 ± 1	190 ± 2	185 ± 3	180 ± 2	176 ± 2	172 ± 3	170 ± 3	172 ± 2	171 ± 2	170 ± 2	170 ± 2	177 ± 2.63**	17.45	08.76
13	195 ± 1	187 ± 2	182 ± 3	176 ± 2	170 ± 2	163 ± 3	160 ± 3	158 ± 2	158 ± 2	159 ± 2	158 ± 2	169 ± 4.03**	25.09	13.33
14	196 ± 1	191 ± 2	186 ± 3	180 ± 2	176 ± 2	171 ± 3	170 ± 3	171 ± 2	170 ± 2	172 ± 2	170 ± 2	177 ± 2.84**	17.18	08.76
15	194 ± 1	187 ± 2	180 ± 3	176 ± 2	172 ± 2	160 ± 3	158 ± 3	158 ± 2	160 ± 2	158 ± 2	158 ± 2	169 ± 4.01**	25.54	12.88
16	195 ± 1	190 ± 2	187 ± 3	182 ± 2	176 ± 2	170 ± 3	168 ± 3	166 ± 2	167 ± 2	166 ± 2	166 ± 2	175 ± 3.29**	19.00	10.25
17	194 ± 1	190 ± 2	185 ± 3	178 ± 2	174 ± 2	172 ± 3	170 ± 3	171 ± 2	170 ± 2	170 ± 2	170 ± 2	176 ± 2.47**	18.00	09.27
18	192 ± 1	187 ± 2	182 ± 3	175 ± 2	167 ± 2	160 ± 3	155 ± 3	155 ± 2	155 ± 2	156 ± 2	155 ± 2	167 ± 4.32**	27.54	13.02
19	195 ± 1	180 ± 2	176 ± 3	169 ± 2	164 ± 2	162 ± 3	162 ± 3	162 ± 2	163 ± 2	162 ± 2	162 ± 2	168 ± 3.23**	25.90	13.84
20	196 ± 1	188 ± 2	182 ± 3	177 ± 2	171 ± 2	166 ± 3	165 ± 3	166 ± 2	165 ± 2	166 ± 2	165 ± 2	173 ± 3.29**	21.36	11.73
21	194 ± 1	185 ± 2	180 ± 3	176 ± 2	170 ± 2	167 ± 3	162 ± 3	162 ± 2	160 ± 2	162 ± 2	162 ± 2	170 ± 3.42**	23.81	12.37
22	195 ± 1	190 ± 2	184 ± 3	178 ± 2	170 ± 2	167 ± 3	167 ± 3	166 ± 2	167 ± 2	166 ± 2	166 ± 2	174 ± 3.25**	20.54	10.76
23	194 ± 1	185 ± 2	179 ± 3	172 ± 2	164 ± 2	160 ± 3	154 ± 3	154 ± 2	155 ± 2	155 ± 2	155 ± 2	166 ± 4.30**	28.63	14.43
24	192 ± 1	170 ± 2	165 ± 3	150 ± 2	145 ± 2	146 ± 3	146 ± 3	145 ± 2	145 ± 2	146 ± 2	146 ± 2	154 ± 4.60**	40.54	19.79
25	195 ± 1	190 ± 2	186 ± 3	180 ± 2	176 ± 2	172 ± 3	172 ± 3	170 ± 2	171 ± 2	170 ± 2	170 ± 2	177 ± 2.72**	17.27	09.23
26	196 ± 1	188 ± 2	182 ± 3	177 ± 2	172 ± 2	166 ± 3	170 ± 3	172 ± 2	170 ± 2	172 ± 2	170 ± 2	176 ± 2.61**	18.27	10.20
27	194 ± 1	184 ± 2	178 ± 3	174 ± 2	170 ± 2	165 ± 3	160 ± 3	162 ± 2	161 ± 2	160 ± 2	161 ± 2	169 ± 3.48**	25.00	12.88
28	195 ± 1	188 ± 2	182 ± 3	177 ± 2	172 ± 2	164 ± 3	160 ± 3	162 ± 2	160 ± 2	160 ± 2	160 ± 2	170 ± 3.85**	23.81	12.82
29	194 ± 1	190 ± 2	183 ± 3	176 ± 2	170 ± 2	166 ± 3	166 ± 3	165 ± 2	166 ± 2	167 ± 2	166 ± 2	173 ± 3.22**	21.18	10.82
30	192 ± 1	187 ± 2	180 ± 3	174 ± 2	170 ± 2	172 ± 3	170 ± 3	170 ± 2	171 ± 2	170 ± 2	170 ± 2	175 ± 2.35**	19.63	08.85
PZN	193 ± 1	180 ± 2	170 ± 3	165 ± 2	150 ± 2	145 ± 3	145 ± 3	146 ± 2	146 ± 2	145 ± 2	145 ± 2	157 ± 5.15**	37.45	18.65
TC	196 ± 2	195 ± 3	194 ± 1	195 ± 2	194 ± 3	195 ± 4	194 ± 2	195 ± 3	193 ± 2	195 ± 2	196 ± 2	194 ± 0.27	—	
CT	100 ± 3	101 ± 1	103 ± 2	105 ± 1	102 ± 2	103 ± 1	101 ± 2	103 ± 3	102 ± 2	101 ± 2	100 ± 2	101 ± 0.45	—	

All values were expressed as Mean ± SEM (*P ≤ 0.05); each group comprised 6 animals (i.e., *n* = 6); MAP (mean arterial pressure); SBP (systolic blood pressure). Prazosin (PZN), toxic control (TC) group was compared with control (CT) group. All the treatment groups were compared with toxic control group and *P* < 0.05 was considered to be significant. **P < 0.01, *P < 0.05, and ^ns^nonsignificant. % inhibition in SBP = initial SBP−MAP/initial SBP ×100.

**Table 2:** Effect of adrenaline alone and after dosing of compounds 1, 16, 19, 20, 23, 24, 27, and 28 on systolic (SABP) and diastolic (DABP) arterial blood pressure and heart rate (HR) of anaesthetized albino rats.

Comp. number	Parameter	Effect of adrenaline alone			Effect of adrenaline after 1/2 h. from inj. test comp.			Effect of adrenaline after 1 h from inj. test comp.			Effect of adrenaline after 2 h from inj. test comp.		
		control	effect	% change	30 min.	effect	% change	60 min.	effect	% change	120 min.	effect	% change
1	SABP	142 ± 4.8	183 ± 3.4*	+28.8	152 ± 1.8	162 ± 2*	+6.5	150 ± 1.9	155 ± 1.2*	+3.3	150 ± 1	150 ± 1.8^ns^	0
	DABP	108 ± 5.3	126 ± 4*	+16.6	104 ± 1.5	114 ± 1.5*	+9.6	100 ± 2.6	98 ± 1.6^ns^	−2	100 ± 1.5	100 ± 1.2^ns^	0
	HR	288 ± 15	365 ± 18*	+21	328 ± 14	330 ± 14.9^ns^	0	328 ± 10	328 ± 8^ns^	0	328 ± 14	328 ± 12^ns^	0

16	SABP	153 ± 4.6	183 ± 3.7*	+19.6	143 ± 2	132 ± 1.6*	−7.6	141 ± 2.5	138 ± 2.4*	−2.1	141 ± 1.8	141 ± 2^ns^	0
	DABP	114 ± 4.2	132 ± 2*	+15.7	92 ± 2	85 ± 1.6*	−7.6	87 ± 1.6	84 ± 1*	−3.4	87 ± 2	87 ± 1.6^ns^	0
	HR	250 ± 18	310 ± 12.5*	+24	260 ± 7	265 ± 8.2*	+1.9	260 ± 11	260 ± 8.2^ns^	0	260 ± 10	260 ± 12^ns^	0

19	SABP	131 ± 2.6	157 ± 5.4*	+19.8	121 ± 2	110 ± 1.7*	−9	120 ± 2	114 ± 1.8*	−5.2	120 ± 1.6	116 ± 2.6*	−3.4
	DABP	104 ± 4.1	130 ± 2.8*	+25	85 ± 1.5	78 ± 1.5*	−8.2	85 ± 1.5	80 ± 1.3*	−6.2	85 ± 2.2	85 ± 2^ns^	0
	HR	255 ± 8	370 ± 12*	+31	372 ± 9^a^	372 ± 12^ns^	0	372 ± 15	372 ± 9^ns^	0	372 ± 12	372 ± 7^ns^	0

20	SABP	150 ± 4.4	188 ± 3.6*	+25.3	100 ± 1.6	90 ± 1.7*	−10	100 ± 2.5	96 ± 1.8*	−4	100 ± 2	100 ± 1.2^ns^	0
	DABP	105 ± 7.7	127 ± 2.5*	+20.9	90 ± 2.3	84 ± 1.3*	−6.6	90 ± 1.2	88 ± 2*	−2.2	90 ± 2.6	90 ± 2.4^ns^	0
	HR	240 ± 8	300 ± 7.8*	+25	263 ± 10	263 ± 7^ns^	0	263 ± 14	263 ± 10^ns^	0	263 ± 12	263 ± 9^ns^	0

23	SABP	150 ± 2.2	191 ± 2.4*	+27.3	102 ± 1.8	92 ± 1.5*	−9.8	100 ± 1.6	86 ± 2.4*	−14	100 ± 1.8	90 ± 2.4*	−10
	DABP	111 ± 2.2	132 ± 1.8*	+18.9	76 ± 1.2	66 ± 1.2*	−13	76 ± 2	70 ± 1.8*	−8.5	76 ± 1.2	72 ± 1.8*	−5.5
	HR	280 ± 12	345 ± 15*	+18.8	328 ± 14	328 ± 14^ns^	0	328 ± 18	328 ± 14^ns^	0	328 ± 14	328 ± 10^ns^	0

24	SABP	141 ± 2.5	181 ± 4.8*	+28.3	86 ± 2	76 ± 1.7*	−11.6	85 ± 2.3	68 ± 2.4*	−20	82 ± 1.6	72 ± 2.1*	−13.8
	DABP	116 ± 2.2	142 ± 4.3*	+22.4	71 ± 2	62 ± 1.5*	−12.6	62 ± 1.8	57 ± 1.6*	−10.7	64 ± 1.4	56 ± 1.3*	−14.2
	HR	268 ± 8	310 ± 10*	+13.5	281 ± 9	281 ± 12^ns^	0	281 ± 18	281 ± 10^ns^	0	281 ± 12	281 ± 8^ns^	0

27	SABP	131 ± 2.6	161 ± 34.4*	+22.9	130 ± 2.9	120 ± 2.3*	−7.6	128 ± 1.7	125 ± 2*	−2.3	128 ± 2.4	128 ± 1.4^ns^	0
	DABP	101 ± 2.6	132 ± 2.9*	+30.6	92 ± 1.5	82 ± 1.5*	−10.8	90 ± 1	88 ± 1.8*	−2.2	90 ± 1.8	90 ± 1^ns^	0
	HR	280 ± 12	365 ± 9*	+23	371 ± 8	272 ± 8^ns^	0	371 ± 9	272 ± 12^ns^	0	371 ± 14	272 ± 8^ns^	0

28	SABP	160 ± 2.7	199 ± 2.4*	+24.3	112 ± 1.6	102 ± 2*	−6.2	110 ± 1.2	112 ± 2*	−1.7	110 ± 2	110 ± 1.5^ns^	0
	DABP	121 ± 2.0	146 ± 1.3*	+20.6	102 ± 1.8	93 ± 2*	−8.8	100 ± 2.5	98 ± 2*	−2	100 ± 1.2	98 ± 2.3	−2
	HR	250 ± 15	288 ± 8*	+23	262 ± 12	264 ± 10^ns^	0	262 ± 12	262 ± 8^ns^	0	262 ± 16	262 ± 10^ns^	0

PZN	SABP	125 ± 0.7	146 ± 2.3*	+16.8	84 ± 1.3	75 ± 1.5*	−10.7	82 ± 2.2	80 ± 1.7*	−2.4	83 ± 2.4	82 ± 2.2*	−1.2
	DABP	102 ± 1.5	121 ± 1.5*	+18.6	65 ± 2	60 ± 1.7*	−7.6	60 ± 2.3	59 ± 2.4^ns^	−1.6	61 ± 1.8	61 ± 1.8^ns^	0
	HR	265 ± 10	320 ± 8*	+20.7	298 ± 12	310 ± 9*	+4	284 ± 13	277 ± 18*	−2.4	273 ± 13	261 ± 12*	−4.5

All values were expressed as Mean ± S.D.; each group comprised 6 animals (i.e., *n* = 6), prazosin (PZN). *Significantly different from respective control value at *P* < 0.05. N.s. = nonsignificantly different from respective control value at *P* < 0.05.

% change = BP initial−BP after drug adm./BP initial ×100.
